# Cytoplasmic Kaiso is associated with poor prognosis in non-small cell lung cancer

**DOI:** 10.1186/1471-2407-9-178

**Published:** 2009-06-09

**Authors:** Shun-Dong Dai, Yan Wang, Yuan Miao, Yue Zhao, Yong Zhang, Gui-Yang Jiang, Peng-Xin Zhang, Zhi-Qiang Yang, En-Hua Wang

**Affiliations:** 1 Department of Pathology, First Affiliated Hospital and College of Basic Medical Sciences of China Medical University, Shenyang 110001, China

## Abstract

**Background:**

Kaiso has been identified as a new member of the POZ-zinc finger family of transcription factors that are implicated in development and cancer. Although controversy still exists, Kaiso is supposed to be involved in human cancer. However, there is limited information regarding the clinical significance of cytoplasmic/nuclear Kaiso in human lung cancer.

**Methods:**

In this study, immunohistochemical studies were performed on 20 cases of normal lung tissues and 294 cases of non-small cell lung cancer (NSCLC), including 50 cases of paired lymph node metastases and 88 cases with complete follow-up records. Three lung cancer cell lines showing primarily nuclear localization of Kaiso were selected to examine whether roles of Kaiso in cytoplasm and in nucleus are identical. Nuclear Kaiso was down-regulated by shRNA technology or addition a specific Kaiso antibody in these cell lines. The proliferative and invasive abilities were evaluated by MTT and Matrigel invasive assay, transcription of Kaiso's target gene matrilysin was detected by RT-PCR.

**Results:**

Kaiso was primarily expressed in the cytoplasm of lung cancer tissues. Overall positive cytoplasmic expression rate was 63.61% (187/294). The positive cytoplasmic expression of Kaiso was higher in advanced TNM stages (III+IV) of NSCLC, compared to lower stages (I+II) (*p *= 0.019). A correlation between cytoplasmic Kaiso expression and lymph node metastasis was found (*p *= 0.003). In 50 paired cases, cytoplasmic expression of Kaiso was 78.0% (41/50) in primary sites and 90.0% (45/50) in lymph node metastases (*p *= 0.001). The lung cancer-related 5-year survival rate was significantly lower in patients who were cytoplasmic Kaiso-positive (22.22%), compared to those with cytoplasmic Kaiso-negative tumors (64.00%) (*p *= 0.005). Nuclear Kaiso staining was seen in occasional cases with only a 5.10% (15/294) positive rate and was not associated with any clinicopathological features of NSCLC. Furthermore, after the down-regulation of the nuclear expresses Kaiso *in vitro*, both proliferative and invasive abilities of three cancer cell lines were significantly enhanced, along with the up-regulation of Kaiso target gene, *matrilysin*.

**Conclusion:**

Our data suggest cytoplasmic Kaiso expression is associated with poor prognosis of NSCLC and various subcellular localizations of Kaiso may play differential biological roles in NSCLC.

## Background

The transcriptional repressor Kaiso belongs to the BTB/POZ (Broad-Complex, Tramtrack and Bric-a-brac/Pox virus, and Zinc finger) family[1,2]. This protein contains an amino-terminal, protein-protein interaction BTB/POZ domain and a carboxyl-terminal DNA-binding C_2_H_2 _zinc finger domain[2]. To date, Kaiso appears to be the only known POZ-ZF transcription factor that possesses bi-modal DNA-binding activity. The candidate Kaiso target genes identified thus far, such as *matrilysin*, *c-myc*, and *cyclin D1*, seem to be regulated via its zinc finger domain[3,4].

However, the role of Kaiso still needs to be defined in tumorigenesis. Considering that some cancer-associated canonical and noncanonical Wnt target gene, such as *matrilysin *and *Wnt11*[3,5], are repressed by Kaiso, it seems that this protein might function as a tumor suppressor. Conversely, data obtained from Kaiso-null mice strongly conflicts with this notion[6]. When Kaiso-deficient mice were cross-bred with the well-characterized, tumor-susceptible *Apc*^Min/+ ^mice, the progeny showed resistance to intestinal tumorigenesis. Furthermore, a recent study carried out in colon cancer cell lines suggests that Kaiso is a methylation-dependent "opportunistic" oncogene, which represses the tumor suppressor gene *CDKN2A *and provides a survival advantage to colon cancer cells[7]. Although controversy still exists, there is no question regarding Kaiso's involvement in human cancer.

To date, little clinicopathological report has referred to the relationship between Kaiso expression and the malignant characteristics of human tumors, including lung cancer. Soubry A. *et al. *initially attempt to explore the expression pattern of Kaiso in human tissues using immunohistochemistry[8]. Interestingly, they found that, in contrast to the nuclear localization of cultured cells (such as MDCK, NIH3T3, HT29, and SW48), this transcription factor predominantly localized to the cytosol in both cancerous and noncancerous human tissues. They also showed that the subcellular localization of Kaiso was dynamic, rather than static, and this phenomenon may contribute to an unexpected influence of the microenvironment. However, further studies are still needed on many topics, including whether this transcription factor exerts a function in the cytoplasm, whether Kaiso is expressed in lung cancer, and the correlation between the subcellular localization of Kaiso and tumor grade and/or prognosis. These issues prompted us to determine the expression profile of Kaiso and to clarify the relationship between Kaiso expression and tumor clinicopathological features in lung cancers, using a large specimen size.

In the current study, we examined the expression of Kaiso in 294 cases of non-small cell lung cancer (NSCLC) and analyzed the correlation between the expression of Kaiso and clinicopathological factors. Meanwhile, Kaiso expression in 50 cases of nodal metastases was probed to investigate differences between primary lung cancers and paired lymph node metastases. In order to obtain prognostic data more quickly, immunohistochemistry was performed on partial lung cancer paraffin embedded tissues from five years ago to determine the expression of Kaiso. The effect of Kaiso on prognosis of the patients with lung cancer was analyzed by inspecting follow-up data. In addition, we ablated Kaiso, which is principally localized in the nuclei of cells, in three lung cancer cell lines to investigate alterations in both *matrilysin *transcription and in the cells, proliferative and invasive abilities, to provide insight into the role of Kaiso in the progression of lung cancers.

## Methods

### Tissue samples

Tumor specimens from 294 patients with NSCLC were obtained between 1998 and 2005 following surgical resection at the First Affiliated Hospital of China Medical University. 20 cases (included in the 294 cases) of tumor and paired non-tumor portion (with >5 cm distance from the primary tumor's edge) of the same case were quickly frozen in liquid nitrogen and maintained at -70°C for protein analysis. Among the 294 cases, the lymph node metastases of 50 patients were available. None of the patients had received radiotherapy, chemotherapy, or immunotherapy prior to tumor excision. Of the patients, 165 are male and 129 are female, creating a 1.87:1 ratio of male to female. Patients' ages at the time of surgery ranged from 35 to 81, with an average age of 57.24 years old. The tumors were classified according to the TNM stage revised by the International Union Against Cancer (UICC) in 2002[9]. All specimens were re-evaluated for diagnosis following the criteria for classification of lung cancer by the World Health Organization (WHO) [10], and 133 squamous cell carcinomas and 146 adenocarcinomas were confirmed. A total of 50 samples (21 squamous cell carcinoma, 23 adenocarcinoma, and 6 large cell carcinoma) with autologous lymph node metastases were used as paired samples to perform immunohistochemical analysis. In addition, immunohistochemistry was completed on 88 cases of primary NSCLC paraffin specimens, excised from February 1998 to October 2007, which had complete follow-up records. This study was conducted under the regulations of the Institutional Review Board of China Medical University. Informed consent was obtained from all enrolled patients prior to surgery.

### Immunohistochemical staining and evaluation

As described previously [11-14], formalin-fixed, paraffin-embedded specimens were cut into 4 μm-thick sequential sections. After dewaxing in xylene and rehydrating stepwise in ethanol, sections were boiled in citrate buffer (pH 6.0) for 105 seconds within an autoclave. Endogenous peroxidase activity and non-specific binding were blocked with 3% H_2_O_2 _and non-immune sera, respectively. Sections were then incubated with primary antibodies overnight at 4°C. Specifically, mouse anti-human Kaiso monoclonal antibody (clone 6F, Upstate, Lake Placid, NY, USA) and goat anti-human Kaiso polyclonal antibody (C-18, Santa Cruz Biotechnology, Inc. CA, USA) were used at concentration of 4 μg/ml. The following day, the staining was followed by incubation with biotinylated secondary antibodies (Maixin Biotechnology, Fuzhou, Fujian, China). The peroxidase reaction was developed with 3, 3'-diaminobenzidine tetrahydrochloride (MaiXin Biotechnology). Counterstaining was done lightly with hematoxylin, and the sections were dehydrated in alcohol before mounting. For the negative control, phosphate-buffered saline (PBS) was used in place of the primary antibodies.

All of the stained sections were assessed by three observers (S.D.D., Y.W. and E.H.W) who had no knowledge of the patients' clinical status. Cases with discrepancies were jointly re-evaluated by the investigators, and a consensus was obtained. The sections were evaluated at low magnification (×100) to identify areas where Kaiso was evenly stained. We counted 400 tumor cells and calculated the percentage of positively staining cells. The proportion of cells exhibiting Kaiso expression was categorized as follows: 0: less than 25%; 1: 26%–50%; 2: 51%–75%; and 3: more than 75%. The staining intensity was categorized by relative intensity as follows: 1(weak); 2 (intermediate) and 3 (strong). The proportion and intensity scores were then multiplied to obtain a total score. To obtain final statistical results, score less than 1 was considered as negative, while scores of 2 or more were considered as positive. Cases were scored nuclear positive when ≥5% of the cells reacted with the anti-Kaiso antibody in the nucleus or in both the cytoplasm and nucleus.

### Cell culture, transfection, and antibody inhibition

The BE1 cell line was established from a human pulmonary giant cell carcinoma (a gift from Dr. Jie Zheng, Medical College of Beijing University, Beijing, China). Human lung adenocarcinoma cell lines LTEP-A-2 and SPC-A-1 were obtained from the Cell Bank of Chinese Academy of Science (Shanghai, China). The cells were cultured in RPMI 1640 medium (GIBCO Inc., Los Angeles, CA, USA), containing 10% fetal calf serum (GIBCO Inc., Los Angeles, CA, USA), 100 IU/ml penicillin (Sigma, St Louis, MO, USA), and 100 IU/ml streptomycin (Sigma).

Three Kaiso shRNA plasmids (RHS1764-9214280, RHS1764-9216302, and RHS1764-9692262) and a control non-silencing pSM2 shRNAmir control plasmid (RHS1707) were purchased from the Open Biosystems Company. The silencing sequences, inserted into the backbone plasmid pSHAG-MAGIC2, were as follows (targeted to NCBI: NM_006777):

1. TGCTGTTGACAGTGAGCG

AGGCAGTTATTAGGAGTGAAATTAGTGAAGCCACAGATGTAATTTCACTCCTAATAACTGCCC

TGCCTACTGCCTCGGA;

2. TGCTGTTGACAGTGAGCG

AGGTCAGAAGATCATTACTTTATAGTGAAGCCACAGATGTATAAAGTAATGATCTTCTGACCC

TGCCTACTGCCTCGGA

3. TGCTGTTGACAGTGAGCG

CGCCGTTACTGTGAGAAGGTATTAGTGAAGCCACAGATGTAATACCTTCTCACAGTAACGGCA

TGCCTACTGCCTCGGA (Bold Codes: showing sense, loop and antisense sequences of these shRNA plasmids).

Transfections were carried out using the Arrest-In™ Transfection Reagent (Open Biosystems, USA), according to the manufacturer's instructions. Transfected cells were harvested and subjected to subsequent assays after a 48 h transfection. Considering the relative effectiveness and stability, the second shRNA plasmid was selected by comparing our pilot experiments.

To further confirm the results obtained from the silencing study, a mouse anti-human Kaiso antibody (mAb 6F, Upstate, Lake Placid, NY) was added into the growth medium, and a final concentration of 100 ng/ml was maintained to the end of the study. The corresponding control groups were treated with mouse anti-human IgG (Beijing Zhongshan Golden Bridge Biotechnology Co. Beijing, China) at 100 ng/ml final concentration.

### Immunofluorescent staining

Immunofluorescent staining was performed as described previously[12,15,16]. Briefly, cells grown on glass coverslips were fixed with ice-cold 100% methanol for 15 minutes at -20°C, followed by permeabilization with 0.2% Triton X-100. Kaiso was detected using two mouse monoclonal antibodies (each at a concentration of 4 μg/ml; 6F and 12H, Upstate, Lake Placid, NY and Santa Cruz Biotechnology, Inc. respectively) and a polyclonal antibody (C-18, Santa Cruz Biotechnology, Inc.), which were applied overnight at 4°C. The primary antibody was followed by incubation with a secondary antibody conjugated to a rhodamine/fluorescein isothiocyanate (FITC)-label, at a dilution of 1:100 (Beijing Zhongshan Golden Bridge Biotechnology Co. Beijing, China). The nuclei were counterstained with propidium iodide (PI, 50 μg/ml, Sigma). The cells were examined with an Olympus IX51 fluorescent microscope (Olympus, Tokyo, Japan), and images were recorded with a CoolPIX 5400 camera (Nikon, Japan).

### RT-PCR analysis

Total RNA was isolated using the TRIzol reagent (Invitrogen). cDNA was prepared using the RNA PCR Kit (AMV) Version 3.0 (TaKaRa Bio Inc., Dalian, Liaoning, China), according to the manufacturer's instructions. The sequences of the primer sets, the linear amplification range, the annealing temperatures and the numbers of the PCR cycles are shown in Table 1. The PCR products were electrophoresed in a 1.5% agarose gel containing 0.1 μg/μl ethidium bromide visualized and analyzed using the BioImaging System (UVP, Upland, CA, USA). A grayscale intensity value was determined for each target band, and normalized to β-actin, to provide a value for the transcriptional level of each gene. Each experiment was repeated 5 times.

**Table 1:** Primer sequences, amplification sizes and annealing temperatures used in RT-PCR.

Primer sequence(5' -> 3')		Amplification range	PCR setting Annealing Number of cycles
Kaiso	TGCCTATTATAACAGAGTCTTT	719–966 (NM_006777.3)	50°C, 40 sec 30 cycles
	AGTAGGTGTGATATTTGTTAAAG		
*Matrilysin*	TCTTTGGCCTACCTATAACTGG	241–660 (NM_002423.3)	53°C, 40 sec 35 cycles
	CTAGACTGCTACCATCCGTC		
β-Actin	AGAGCTACGAGCTGCCTGAC	797–1096 (NM_001101.3)	55°C, 40 sec 30 cycles
	AGTACTTGCGCTCAGGAGGA		

### Immunoblotting assay

As described previously[15], frozen tissues (including tumor and non-tumorous portion) or cells were washed twice with ice-cold phosphate buffered saline (PBS), homogenized on ice in 10 volumes(w/v) of lysis buffer containing 20 mM Tris – HCl, 1 mM EDTA, 50 mM NaCl, 50 mM NaF, 1 mM Na3VO4, 1% Triton-X100 and 1 mM PMSF using a homogenizer (Heidoph, DLA ×900). The homogenate was centrifuged at 15000 rpm for 30 min at 4°C. The supernatant was collected and determined protein content by the BCA assay (BCA protein assay kit-23227, Pierce Biotechnology). From each sample preparation, 80 μg of total protein was separated by 8% SDS-PAGE and then transferred to PVDF blotting membranes. The total protein extracts were analyzed by immunoblotting with indicated antibodies following SDS-PAGE analysis. Immunoblots were performed using goat polyclonal primary specific for Kaiso and β-actin (a housekeeping protein used as a loading control to assure equal amounts of protein in all lanes). After blocking non-specific binding with 5% BSA in TBS (pH 7.5) containing 0.05% Tween-20 (TBST), primary antibodies were incubated on the membranes for Kaiso (1:1000, C-18, Santa Cruz Biotechnology, Inc.) and β-actin (1:200, Beijing Zhongshan Golden Bridge Biotechnology Co. Beijing, China) overnight at 4°C. Following three times washes in TBST, the membranes were incubated for 2 h at 37°C with secondary antibodies (1:2000, ZDR-5306) labeled with horseradish peroxidase (all from Zhongshan Biotechnology). Immunoreactive straps were identified using the DAB system (DAB kit-0031, Maixin Biotechnology), as directed by the manufacturer. The BioImaging System (UVP, Upland, CA, USA) was used to catch up the specific bands, and the optical density of each band was measured using Image J software. The ratio between the optical density of interest proteins and β-actin of the same sample was calculated as relative content and expressed graphically.

### 3-(4, 5-Dimethylthiazol-2-yl)-2,5-Diphenyltetrazolium Bromide (MTT) Assay and Matrigel Invasive Assay

The shRNA-Kaiso cells, the Kaiso antibody addition cells, and the control cells were seeded at a density of 5000 cells/well in 96-well plates. Cell proliferation was evaluated each day for four days after the MTT treatment. The absorbance, which is directly proportional to the number of living cells in the culture, was measured at 570 nm using a microplate reader (Model 550, Bio-Rad, Hercules, CA, USA). A blank with dimethyl sulfoxide (DMSO) alone was taken and subtracted from all values.

The cells' invasive abilities were examined using a 24-well Transwell with 8-μm pore polycarbonate membrane inserts (Corning Inc., Corning, NY, USA) according to the manufacturer's protocol. To the upper surface of the membranes, 100 μl Matrigel (1:4 dilution) was applied. After solidification of the Matrigel, 100 μl of the cell suspension (5 × 10^5 ^cells/ml) was added to the upper chamber. Medium supplemented with 10% FBS was added to the lower chamber as the chemoattractant. After incubation for 48 h, the filters were fixed with cold methanol, and the non-invading cells on the upper surface were removed by scrubbing with a cotton swab. The filters were then subjected to hematoxylin staining. Cells that appeared on the lower surface of the filter were counted in five random 200× fields using an inverted microscope (Olympus 1 × 51, Olympus America Inc., Melville, NY, USA). The experiments were performed in triplicate and were repeated three times independently.

### Statistical analysis

The Pearson's Chi-Square test was used to analyze the relationship between cytoplasmic expression of Kaiso and clinicopathological factors. Comparison of cytoplasmic Kaiso expression between primary tumors and lymph node metastases was accomplished using the McNemar's test. All data were expressed as mean ± standard deviation (S.D.) for *in vitro *experiments and were performed at least three times. The probabilities of overall survival were calculated using the Kaplan-Meier method and were compared using the log-rank test. For determining factors related to overall survival, a Cox proportional hazard model was utilized. All statistical analyses were performed using SPSS 13.0 for Windows (SPSS Inc., Chicago, IL, USA). *p*-values less than 0.05 were considered statistically significant.

## Results

### Kaiso was expressed in the cytoplasm of lung cancer cells and is associated with the malignancy of NSCLC

**Figure 1:**
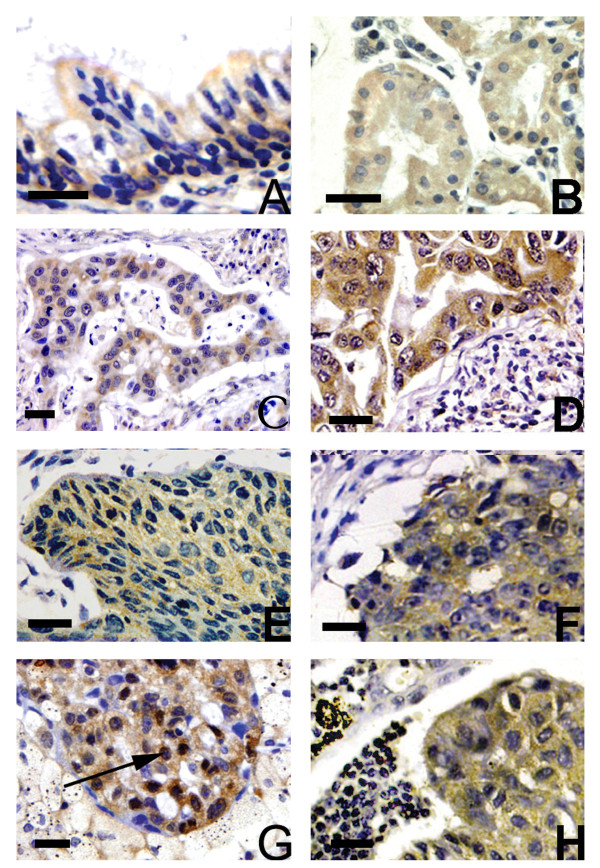
**Immunohistochemical analysis of Kaiso expression**. Kaiso was expressed in the cytoplasm of normal adult bronchial epithelial cells (A) and glands (B). The expression of Kaiso was increased in metastases of lung adenocarcinomas (D) and squamous cell carcinomas (F), compared to the matched primary tumor (C, D). Nuclear Kaiso staining was observed occasionally primary tumors (G, black arrow), with cytoplasmic staining of tumor cells in paired metastases. A magnification scale bar of 20 μm is shown.

Kaiso was weakly expressed in the ciliated epithelial cells of bronchus from all 20 normal pulmonary tissues and primarily localized on the apiculus of these cells (Figure 1A) and several glands (Figure 1B). According to our evaluation criteria, they were judged as negative expression. Positively staining tumor cells primarily showed cytoplasmic labeling of Kaiso (Figure 1C, D, E, and 1F). Nuclear staining was seen in occasional tumor cells but only with a 5.10% (15/294) positive expression rate (Figure 1G). This pattern of staining was not associated with any clinicopathological features of NSCLC (data not shown). The specificity of the Kaiso subcellular staining was confirmed with another polyclonal antibody (C-18).

The positive cytoplasmic expression of Kaiso in NSCLC was 63.61% (187/294), which is significantly higher than that in normal bronchial epithelium (*p *< 0.001). The relationships between the cytoplasmic expression of Kaiso and the different clinicopathological factors are shown in Table 2. The positive cytoplasmic expression of Kaiso was higher in stages III+IV NSCLC than in stages I+II (*p *= 0.019). In 163 cases with lymph node metastases, 116 (71.2%) showed cytoplasmic expression, and 47 (28.8%) had no cytoplasmic expression. In 131 samples without lymph node metastasis, 71 (54.2%) showed cytoplasmic expression, and 60 (45.8%) had no cytoplasmic expression. There was a significant correlation between cytoplasmic Kaiso expression and lymph node metastasis (*p *= 0.003). In addition, there were no significant correlations between cytoplasmic Kaiso expression and gender, age, differentiation, or histology.

**Table 2:** Relationship between cytoplasmic Kaiso expression and clinical/histological features in 294 patients with NSCLCs

Variables	All patients	Cytoplasmic Kaiso expression		p*
			
		Negative	Positive	
**Total**	294	107	187	
**Age(y)**				
≤ 55	125	51	74	0.177
>55	169	56	113	
**Gender**				
Male	165	59	106	0.797
Female	129	48	81	
**Stage**				
I/II	152	65	87	0.019
III/IV	142	42	100	
**Histology**				
Squamous cell Carcinoma	133	41	92	0.343
Adenocarcinoma	146	60	86	
Large cell Carcinoma	12	5	7	
Adenosquamous Carcinoma	3	1	2	
**Grade**				
Well	78	33	45	0.296
Moderate	100	36	64	
Poor	116	38	78	
**Lymph node metastasis**				
Yes	163	47	116	0.003
No	131	60	71	

*p values were obtained with the X^2 ^test (two-sided).

**Well, moderate, versus poor.

The expression of Kaiso in the 50 cases for which paired data were available is summarized in Table 3. The positive scoring of cytoplasmic Kaiso was 78.0% (39/50) in the primary sites and 90.0% (45/50) in the lymph node metastases. Lymph node metastases showed an increased expression rate in cytoplasmic Kaiso, compared to the primary tumors (*p *= 0.001).

**Table 3:** Correlation between cytoplasmic Kaiso expressions in matched primary tumors and autologous lymph node metastases of NSCLCs

		Cytoplasmic Kaiso in the Primary Growth		Total	*p*
		
		Positive	Negative		
**Cytoplasmic Kaiso in** **Lymph node metastases**	**Positive**	38	7	45	0.001
	**Negative**	1	4	5	
	**Total**	39	11		

**Figure 2:**
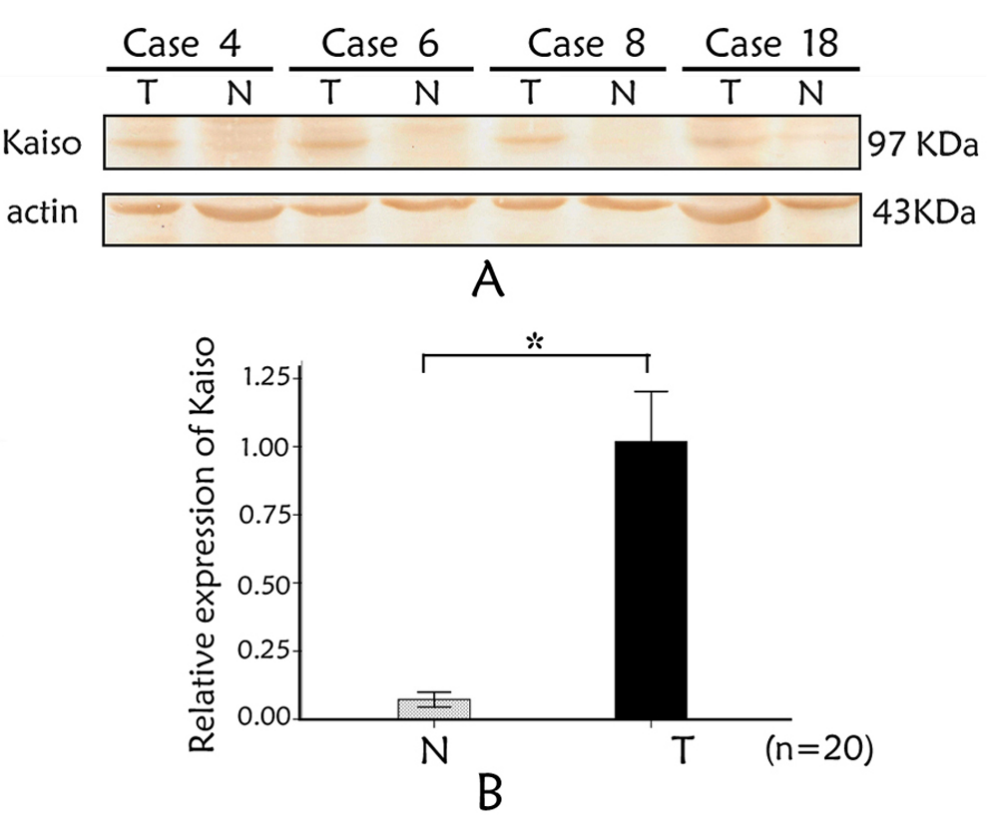
**Kaiso expression in NSCLC by Western blotting**. (A) Representative results of Kaiso protein expression in matched tumourous (T) and surrounding non-tumorous (N) tissues from 8 of 20 NSCLC patients. Lane T: tumor tissue; Lane S: Surrounding normal lung tissue. Samples: case 4; case 6; case 8; case 18. Band intensities indicate significant Kaiso up-regulation in tumorous in comparison with the non-tumorous tissue of the same patient. β-actin was used as a loading control to assure equal amounts of protein in all lines. (B) The ratio between the optical density of Kaiso and β-actin of the same patient was calculated and expressed graphically. The significant difference of Kaiso expression between tumorous (T) and non-tumorous (N) tissues was analyzed statistically. Kaiso immunoreactivity is greater in neoplastic tissues (*p *= 0.000). Data were expressed as mean ± standard deviation (S.D.). Columns, mean (n = 20); error bars, S.D.

Western blotting was used to evaluate Kaiso expression in 20 NSCLC and paired non-tumorous lung tissues distant from the primary tumor of the same case. The increased Kaiso expression was found in 18 NSCLC samples in comparison with the non-tumorous counterparts. The western blotting of four samples is shown in Figure 2A, and the optical density of the tumorous (T) and non-tumorous (N) tissues of the same patient was measured and expressed graphically (Figure 2B). Kaiso expression was significant higher in tumorous tissues (t = 10.610, n = 20, *p *= 0.000).

**Figure 3:**
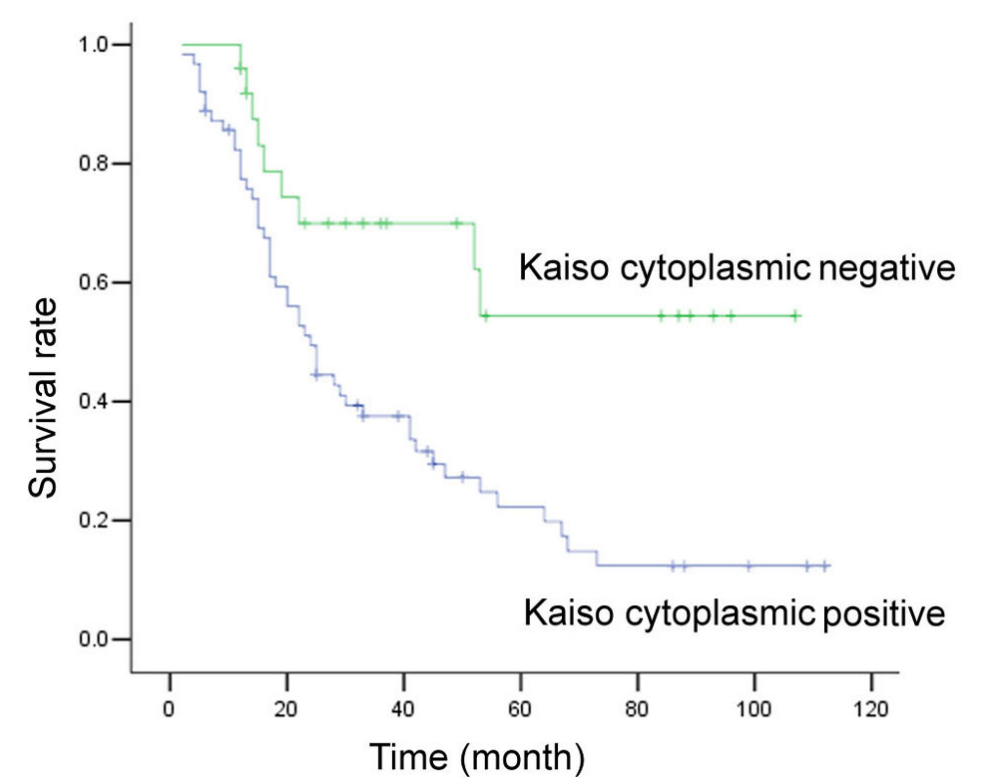
**Kaplan-Meier analysis showing overall survival among NSCLC patients, based on their positive and negative Kaiso expression**. Positive cytoplasmic expression of Kaiso was significantly correlated with poor prognosis (p = 0.002).

The overall Kaplan-Meier survival curves for cytoplasmic Kaiso expression are shown in Figure 3. The total lung cancer-related five-year survival rate was 34.1%, while 22.22% in patients positive for cytoplasmic Kaiso and 64.00% in patients negative for cytoplasmic Kaiso. Univariate analysis revealed cytoplasmic expression of Kaiso to be linked to poor overall survival. Survial rate of patients with positive cytoplasmic Kaiso expression was significantly lower than those with cytoplasmic Kaiso-negative tumors (*p *= 0.002, Figure 3).

To further evaluate the cytoplasmic expression of Kaiso as prognostic factor, a multivariate Cox regression analysis was carried out. As shown in Table 4, in an analysis of 88 patients, lymph node metastasis (*p *= 0.001) and tumor stage (*p *= 0.047) were independent prognostic factors. Additionally, cytoplasmic Kaiso status may be an independent prognostic factor for the p-value (*p *= 0.054).

**Table 4:** Multivariate Cox proportional hazard analysis for overall survival of 88 patients with NSCLCs

Factors	β	S.E.	*P *value	Exp (β)	95% CI for Exp (β)
**TNM stage**	0.487	0.245	0.047	1.627	1.007–2.632
**Lymph node metastasis**	1.726	0.507	0.001	5.620	2.079–15.196
**Cytoplasmic Kaiso expression**	0.718	0.372	0.054	2.051	0.988–4.256

### shRNA-Kaiso effectively ablated nuclear Kaiso expression of in vitro cultured lung cancer cells

**Figure 4:**
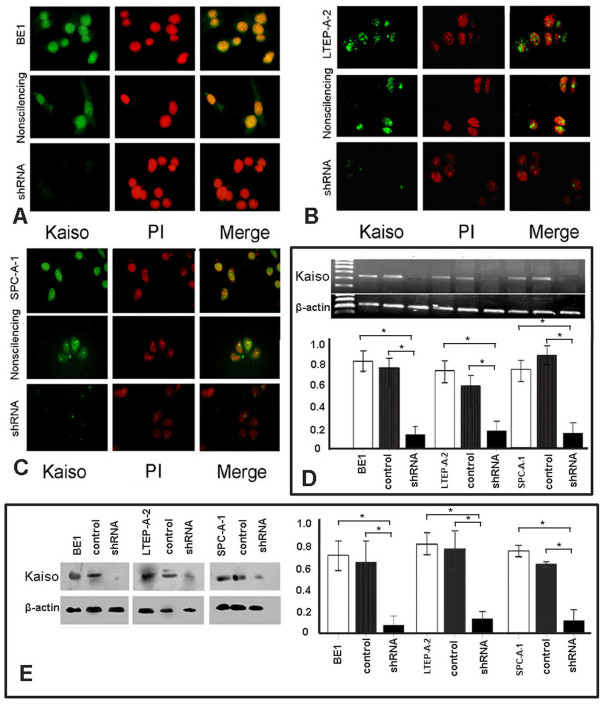
**shRNA-Kaiso efficiently down-regulates nuclear Kaiso expression in three lung cancer cell lines**. shRNA-Kaiso efficiently down-regulated nuclear Kaiso expression in three lung cancer cell lines. Specifically, nuclear staining of Kaiso was detected by immunofluorescence in BE1, LTEP-A-2, and SPC-A-1 cells (top rows in A, B, and C). After transfected with shRNA-Kaiso, BE1, LTEP-A-2, and SPC-A-1 cells showed significantly reduced green/yellow signals in the nucleus, while there was no signal detected in several transfected cells (bottom rows in A, B, and C), compared with controls (middle rows in A, B, and C). Results from RT-PCR and immunoblotting assays were shown in D and E. Little bands or dots can be detected after transfection with shRNA-Kaiso, which demonstrated that levels of Kaiso mRNA and protein were down-regulated significantly (*p *< 0.05). β-actin served as an internal control. Data were expressed as mean ± standard deviation (S.D.). Columns, mean (n = 3); error bars, S.D.

In contrast to the cytoplasmic localization pattern of Kaiso in tissues, all three lung cancer cell lines showed a primarily nuclear localization of Kaiso. Following transfection with shRNA-Kaiso, the nuclear Kaiso staining was accordingly decreased, even vanishing in several transfected lung cancer cells, which was not observed in the control cells (Figure 4A, B, and 4C). In addition, RT-PCR and immunoblotting results demonstrated that Kaiso mRNA and protein levels were significantly down-regulated in the shRNA-Kaiso cells, compared with the control group (*p *< 0.05 in all lung cancer cell lines).

### Down-regulating nuclear Kaiso increases matrilysin transcription and enhances the proliferative and invasive abilities of lung cancer cells

**Figure 5:**
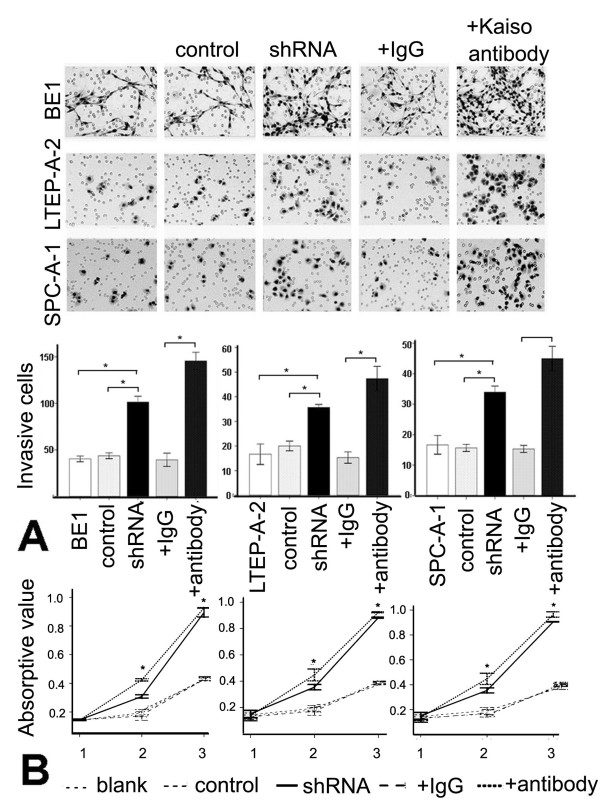
**Down-regulated nuclear Kaiso increases matrilysin transcription and enhances the proliferative and invasive ability of lung cancer cells**. A. Invasion assays of BE1, LETP-A-2, and SPC-A-1 cells, which were transfected with shRNA-Kaiso or treated with the addition of a Kaiso antibody. Forty-eight hours after plating on Matrigel, the number of cells invading the lower surface of the filter was far greater in the controls. Data were expressed as mean ± standard deviation (S.D.). Columns, mean (n = 3); error bars, S.D. B. Cell proliferation was determined using the MTT assay. The absorbance at 570 nm represents cell viability at each time point and is a measure of cell proliferation. The growth rates were not markedly different during the first day after transfection, but in each of the latter two days, the growth rate was significantly higher in shRNA-Kaiso groups, compared to controls. For the Kaiso antibody addition groups, significant differences were observed at all three days. Points, mean (n = 3); Bar, S.D. *, *p *< 0.001.

Since Kaiso primarily localized to the nucleus *in vitro*, it was conceivable to explore the biological role of nuclear Kaiso in lung cancers using *in vitro *cultured cells. The MTT assay results demonstrated that after down-regulating nuclear Kaiso by transfecting shRNA-Kaiso, the levels of proliferation were significantly higher in the shRNA-Kaiso group cells, compared to the control cells [*p *> 0.05(day 1); *p *< 0.05(day 2–3), n = 3] (Figure 5B). For the Kaiso antibody addition groups, the growth rates were markedly different from the control cells at all three days. Meanwhile, the shRNA-Kaiso cells and the Kaiso antibody-treated cells showed increased invasion onto the lower surfaces of the Transwell filters, compared to control cells (*p *< 0.01, Figure 5A).

**Figure 6:**
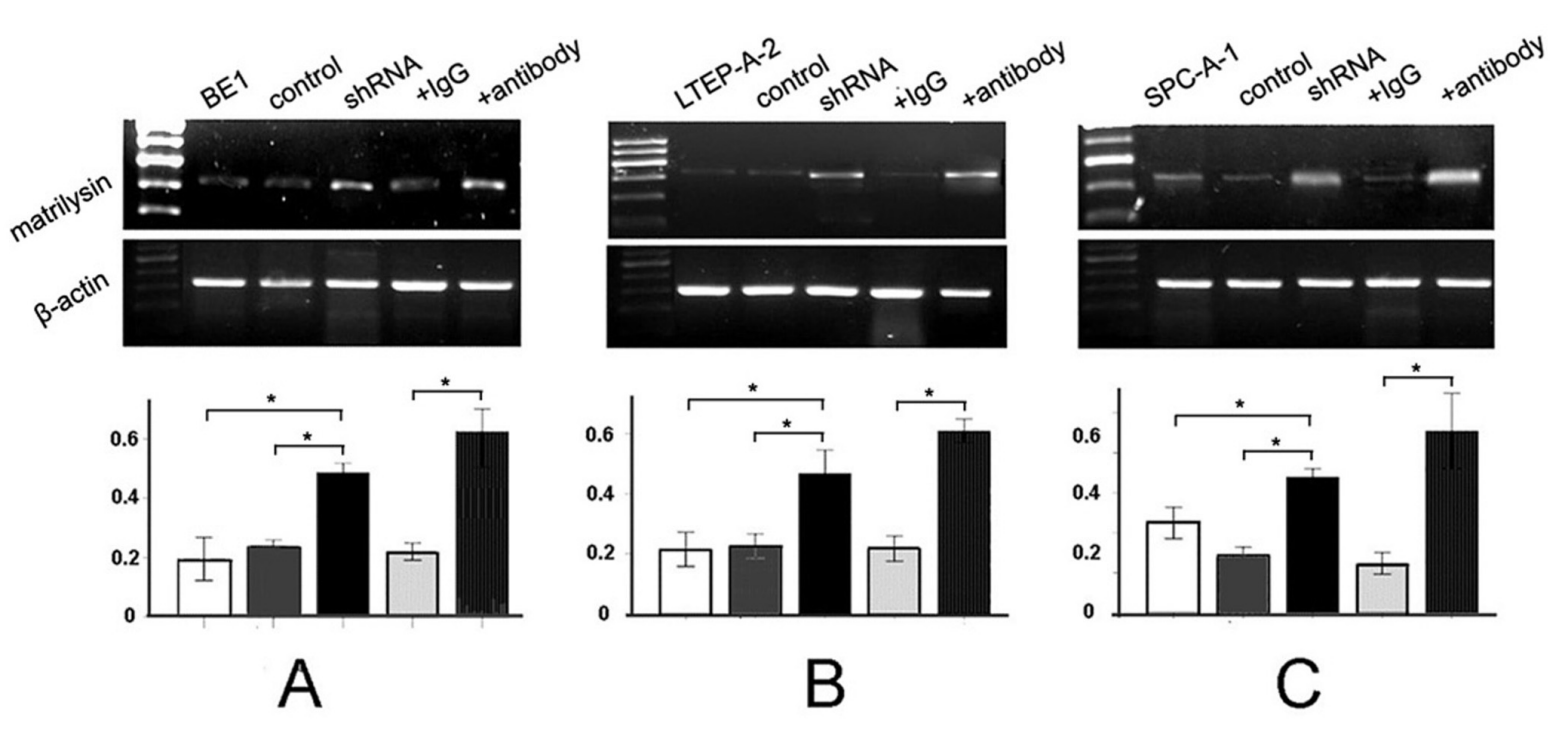
**Down-regulated nuclear Kaiso increases matrilysin transcription in three kinds of lung cancer cell lines**. (A) The electrophoresis images of *matrilysin *mRNA in BE1, BE1+empty, BE1+shRNA-Kaiso, BE1+IgG, and BE1+Kaiso antibody cells after RT-PCR. β-actin served as an internal control. The relative expression of *matrilysin *mRNA was significantly enhanced in BE1+shRNA-Kaiso and BE1+Kaiso antibody cells. Columns, mean (n = 3); bar, SD. *, *p *< 0.01. (B) The electrophoresis images of *matrilysin *mRNA in LTEP-A-2, LTEP-A-2+empty, LTEP-A-2+shRNA-Kaiso, LTEP-A-2+IgG, and LTEP-A-2+Kaiso antibody cells after RT-PCR. β-actin served as an internal control. The relative expression of *matrilysin *mRNA was significantly enhanced in LTEP-A-2+shRNA-Kaiso and LTEP-A-2+Kaiso antibody cells. Columns, mean (n = 3); bar, SD. *, *p *< 0.01. (C) The electrophoresis images of *matrilysin *mRNA in SPC-A-1, SPC-A-1+empty, SPC-A-1+shRNA-Kaiso, SPC-A-1+IgG, and SPC-A-1+Kaiso antibody cells after RT-PCR. β-actin served as an internal control. The relative expression of *matrilysin *mRNA was significantly enhanced in SPC-A-1+shRNA-Kaiso and SPC-A-1+Kaiso antibody cells. Columns, mean (n = 3); bar, SD. *, *p *< 0.01.

To further confirm whether the enhancement of proliferative and invasive abilities contributes to Kaiso down-regulation in the nucleus, *matrilysin *mRNA levels were detected by RT-PCR. The results demonstrated that *matrilysin *mRNA increased significantly in both shRNA-Kaiso and Kaiso antibody-treated cells, compared with controls (*p *< 0.01, Figure 6).

## Discussion

With immunohistochemical analysis of small sample sizes, Kaiso has been shown to be expressed in human tumors, such as breast cancers and prostate cancers, with varying expression in one report[8]. Due to lack of information regarding to Kaiso expression in tumors, no Kaiso positive criterion has been detailed by researchers. Thus, a criterion still needed to be specified in the current study. Based on the basic expression profile of Kaiso in NSCLC and in consulting with general criterion widely applied in immunohistochemical studies, we considered expression of cytoplasmic Kaiso to be positive when scores of 2 or more, because the distribution ratio of stained cells showed bipolarity using this method. We also found that no significant difference between cytoplasmic Kaiso localization and clinicopathological features could be elucidated when other positive criterions were applied.

The transcriptional repressor Kaiso belongs to the BTB/POZ (Broad-Complex, Tramtrack and Bric-a-brac/Pox virus, and Zinc finger) family[1,2], which is abbreviated as POZ-ZF. Many members of the POZ-ZF family have roles in the development of cancer. For example, APM-1 inhibits the growth of cervical carcinoma[17], and the human BCL-6 and promyelocytic leukemia zinc finger proteins are causally involved in non-Hodgkin's lymphoma and acute promyelocytic leukemia, respectively[18,19]. In addition, Pokemon was proven to be a type of proto-oncogene[20], whereas the BTB/POZ protein HIC1 is candidate tumor suppressor in a variety of human tumors [21-23]. However, the relationship between Kaiso and cancer still needs to be clarified. Some data clearly indicate a tumor-suppressor activity[3,24], while the fact that a Kaiso knockout was detrimental to tumor growth and survival in an animal model suggests that Kaiso facilitates tumorigenesis[6]. The latter point is consistent with results obtained from a recent study where Kaiso depletion sensitized colon cancer cells to cell cycle arrest and chemotherapy.

There is little documentation regarding to the immunohistochemical expression of Kaiso in lung cancer. Only one report examines Kaiso expression in lung cancer tissues, and the author reported no Kaiso expression in two lung squamous cell carcinomas[8]. Since there is little current knowledge about Kaiso expression in lung cancer, its expression profile and relationship to clinical characteristics still needed to be clarified. In the present study, we examined the expression of Kaiso in 294 cases non-small cell lung cancers (NSCLC) and analyzed the correlation between expression of Kaiso and clinicopathological factors. Meanwhile, Kaiso expression was assessed in 50 cases of lymph node metastases to investigate differences between primary lung cancer and paired lymph node metastases. Our study demonstrated that 63.61% of 294 lung cancer samples contained cytoplasmic Kaiso expression, which is a significant increase compared to normal bronchial epithelial cells (regarded as negative expression). This data implied an oncogenic role for Kaiso. Besides, the 294 cases with primary lung cancer showed that, cytoplasmic Kaiso expression in lung cancer tissue of patients with TNM stages III+IV was significantly higher than that in TNM stages I+II (*p *= 0.019). Moreover, cytoplasmic expression (71.2%) of Kaiso in the lung cancer samples from patients with lymph node metastases was significantly higher than that (54.2%) in samples from patients without lymph node metastases, suggesting that cytoplasmic Kaiso expression in primary cells was closely associated with tumor lymph node metastases (*p *= 0.003). In 50 paired cases, we also observed that lymph node metastases had increased (90.0%) cytoplasmic expression of Kaiso, compared to the primary tumors (78.0%) in fifty paired lung cancer specimens. In order to define the effect of cytoplasmic Kaiso on prognosis of the patients with lung cancer, eighty-eight NSCLC tissues with complete follow-up records were analyzed with immunohistochemistry. Prognostic analysis were performed on the clinical information by combining follow-up data, and the results indicate that the postoperative survival period of the group with positive cytoplasmic Kaiso expression was notably shorter than that of the negative group. Specifically, these results suggested that cytoplasmic Kaiso expression was a harmful factor affecting prognosis and further indicated that cytoplasmic Kaiso correlated with malignant tumor behavior. Cox model multivariate analysis showed that cytoplasmic Kaiso may be an independent factor affecting prognosis, with a p-value of 0.054. It seems important to collect more patient follow-up records to clarify this correlation between Kaiso expression and a patient's clinical response. Kaiso may exert an anti-oncogenic function in the cytoplasm of lung cancer cells. Obviously, this suggestion seems antagonistic of its role as a transcriptional repressor, but Kaiso may function in different biological roles in the cytoplasm and the nucleus. While we are still unsure of how Kaiso exerts its function in the cytoplasm, we believe that Kaiso plays a biological role in the cytoplasm, and this role may differ from that in the nucleus.

In lung cancer tissues, we found Kaiso to be primarily localized in the cytoplasm rather than the nucleus. In fact, the positive nuclear scoring of Kaiso was extremely low. Even when we defined nuclear staining of 5% of the cells in a sample as positive, only 15 cases were included. Our statistical analysis showed that nuclear expression of Kaiso did not correlate with various pathological factors. Considering the influence of unexpected tumor microenvironment[8], which may promote Kaiso to translocate from nucleus to cytoplasm, we supposed it was hard to clarify the nuclear role of Kaiso in lung cancer tissues.

Consistent with previous study[8], we also found Kaiso principally localized in the nucleus when cells cultured *in vitro*. The subcellular localization difference of Kaiso between *in vitro *and *in vivo *could be explained by the tumor microenvironment. We also have data implying that other factors, such as the cell cycle and the influence of p120ctn, influence the subcellular localization of Kaiso (data not shown). We did not plan to extend this theme further in present study, although we were interested in whether Kaiso exerted its varying functions in the cytoplasm or the nucleus. Thus, we cultured three kinds of lung cancer cells (BE1, LTEP-A-2, and SPC-A-1)*in vitro *and observed where Kaiso is localized. Indirect immunofluorescence demonstrated that Kaiso is localized to the nucleus in these three lung cancer cell lines. We performed the shRNA technique to down-regulate nuclear localized Kaiso, and we utilized a specific Kaiso antibody as a control. The results demonstrated that both the shRNA-Kaiso and the specific Kaiso antibody addition were able to enhance the proliferative and invasive abilities of lung cancer cell lines.

In order to determine whether the enhancement of proliferative and invasive abilities contributed to the down-regulation of nuclear Kaiso, we analyzed mRNA expression of the *matrilysin *gene, which is directly repressed by Kaiso. This repression is due to the *matrilysin *promoter, which contains two conserved copies of the Kaiso binding sequence (KBS). At present, this gene has been proven to be regulated by Kaiso[3,25]. Addition of specific Kaiso antibodies also relieved the Kaiso-mediated repression of *matrilysin*. These studies demonstrated that the enhancement of proliferative and invasive abilities was consistent with down-regulation of Kaiso in nucleus.

It should be noted that differences in the up-regulation of *matrilysin *transcription and in the proliferative and invasive abilities existed between the Kaiso antibody addition group and the shRNA-Kaiso group. These results could be explained by the fact that different experimental treatments were used on the cells for each experiment; cells transfected with shRNA Kaiso plasmids suffered from the effectiveness of transfection, while treating with a Kaiso antibody could exert more extensive effects throughout the culture.

## Conclusion

Above all, in lung cancer tissues, we found that Kaiso is principally expressed in the cytoplasm, which positively correlated with TNM stage, lymph node metastases, and potentially with unfavorable prognosis of patients. However, whether Kaiso can be used as an independent prognostic factor still needs further clarification (p = 0.054, Cox multivariate analysis). As for several *in vitro *cultured lung cancer cell lines, Kaiso localized primarily in the nucleus. Performing both shRNA-Kaiso and using addition of Kaiso-specific antibodies, we were able to increase the expression of *matrilysin*, which is repressed by Kaiso, and promote proliferative and invasive abilities of the cells. Although our research is still preliminary, it has already demonstrated unique biological functions for the different localization of Kaiso in the cytoplasm and the nucleus. We also noticed that although Kaiso is basically localized to the nucleus in cultured cells, cells showing cytoplasmic or nuclear/cytoplasmic co-expression existed. Thus, it is worthwhile in the future to construct plasmids that enhance the cytoplasmic-specific and nuclear-specific localization of Kaiso in order to clarify the biological roles of cytoplasmic and nuclear Kaiso. These types of studies will also help elucidate the specific mechanisms that will further reveal the inner relationship between cytoplasmic Kaiso expression and lymph node metastasis, as well as poor prognosis.

## Abbreviations

NSCLC: non-small cell lung cancer; KBS: Kaiso binding sequence; MTT: 3-(4,5-Dimethylthiazol-2-yl)-2, 5-diphenyltetrazolium bromide.

## Competing interests

The authors declare that they have no competing interests.

## Authors' contributions

DSD, WY and WEH designed research, evaluated immunohistochemical results and wrote the paper, MY and ZY carried out prepared pathology samples, follow-up and immunohistochemical study, ZY and JGY carried out cell culture, IF, MTT and invasive assay, ZPX and YZQ performed the statistical analysis of all data. The manuscript has been read and approved by all the authors.

## Pre-publication history

The pre-publication history for this paper can be accessed here:

http://www.biomedcentral.com/1471-2407/9/178/prepub
